# The History and Capabilities of Herbal Simples

**Published:** 1892-09-03

**Authors:** W. T. Fernie


### The History and Capabilities of Herbal Simples.

By W. T. Fernie, M.D.

(*Continued from page 336.*)

### GARLIC AND THE ONION.

Among the lilies of the field which toil not, neither do they spin, but yet are clothed more grandly than Solomon in all his glory, may be truly classified the onion, the leek, and the garlic. Their Scriptural mission is followed likewise to the end, since, whilst growing handsomely to-day, they are cast to-morrow into the oven for the benefit and comfort of mankind. These severally belong to the genus allium, each containing allyl, which is a radical rich in sulphur. They possess the same common properties and characteristics, but in different degrees. The homely onion may be taken as the best illustration of the tribe. This is named Allium cepa—from cep, a head (of bunched florets which it bears). Lucilius calls it "flebile cœpe," because the pungency of its odour will provoke a flow of tears from the eyes; and Shakespeare says about it in "Taming of the Shrew":—

> "If the boy have not a woman's gift
> To rain a shower of commanded tears
> An onion will do well."

And again:

> "Mine eyes smell onions;
> I shall weep anon."

The Egyptians were devoted to onions, which they ate more than two thousand years before the time of Christ. They were given to swear by the onion and the garlic in their gardens. Herodotus tells us that during the building of the Pyramids nine tons of gold were spent in buying onions for the workmen. But it is to be noted that in Egypt the onion is sweet and soft; whereas in other countries it grows hard, and nauseous, and strong. By the Greeks and the Romans it was thought to be efficacious against witches, because respected by the devil, from its being an object of worship as well as himself. In some places, as at Torquay, it is customary even now to throw an onion after a bride, so as to keep away the powers of evil. That the onions of the Egyptians were identical with those of our time has been proved by growing recently in the ordinary way a bulb taken from the hand of a primeval mummy. In Bohemia the onion is used for fortune telling. It is thought if hung up in a room to draw to itself maladies which would otherwise fall on the inmates. During our last epidemic of cholera in England, it puzzled the sanitary inspectors of a northern town to understand why the inhabitants of one cottage in a certain row were not affected by the disease which was raging among their neighbours. Finally it became noticed that a net of onions was suspended in the fortunate house, and on examination these were all found to be diseased. It is also related that during a former outbreak of infectious fever in Somers Town and St. Giles's, the French priests, who constantly used garlic in all their dishes, visited the worst cases in the dirtiest hovels with impunity, while the English clergy who were similarly engaged, but who did not eat onions in like fashion, caught the infection in many instances, and fell victims to the disease. Raw onions contain an acrid volatile oil, sulphur, phosphorus, alkaline earthy salts, starch, and free, uncrystallised sugar. The fresh juice is colourless, but by exposure to the air becomes red. This juice anointed upon a bald head in the sun bringeth the hair again very speedily. A syrup made from the juice with honey is an excellent medicine for old phlegmatic persons in cold weather, when their lungs are stuffed and their breathing is hindered. Raw onions increase the flow of urine, and promote perspiration, insomuch that a diet of these with bread has many a time cured dropsy supervening after a chill or exposure to cold. In making curative simples the onion and garlic should not be boiled, else the volatile essential oil on which their virtues chiefly depend will escape during the process. Their principal internal effects are stimulation and warmth, so that they are better adapted for use when the person is of a cold temperament, and when the vital powers are inclined to be feeble than when the body is feverish and the constitution ardently irritable. "They be naught," says Gerarde, "for those that be cholericke; but good for such as are replete with raw and phlegmatick humors." Onions when eaten at night by those who are not feverish will promote sleep and induce perspiration. "The late Frank Buckland confirmed this statement. He said, "I am sure the essential oil of onions has soporific powers. In my own case it never fails. If I am much pressed with work, and feel that I am not disposed to sleep, I eat two or three small onions, and the effect is magical."

When sliced and applied externally, the raw onion serves by its pungent essential oil to quicken the circulation and to redden the skin of the particular surface; very usefully so in the case of an unbroken chilblain or to counteract pain; but in this crude state the bulb is not emollient or demulcent. If employed as a poultice for earache or broken chilblains the onion should be roasted so as to modify its acrid oil. When there is a constant and painful discharge from the ear of fetid matter, or where an abcess is threatened with pain, heat, and swelling, a hot poultice of roasted onions will be found very useful and soothing. It was generally supposed in the west of Scotland that a poultice of peeled onions laid on the stomach or underneath the armpits would relieve any one that had taken poison. Also that a certain cure for deafness may be found in ants' eggs mixed with the juice of onions and dropped into the ear. So nutritious does the Highlander find the onion that if having a few raw bulbs and a crust of bread or oatcakes in his pocket, he can travel for two or three days together without any other sort of food. Spanish onions, which are imported into this country in the winter, are sweet and mucilaginous. A peasant in Spain will munch an onion just as an English labourer eats an apple. At the present day Egyptians take onions roasted, and each cut into four pieces, with small bits of baked meat, which the Turks call Kebab. With this sweet and savoury dish they are so delighted that they hope to enjoy it in Paradise. The juices of a sliced raw onion are alkaline, and will quickly relieve the acid venom of a sting from a bee or a wasp, if applied promptly to the part. A tincture is made by the chemists from large red, strong onions for medicinal purposes. As a warming expectorant in chronic bronchitis, or asthma, or for a cold, which is not of a highly feverish character, from half to one teaspoonful of the tincture may be given with benefit three or four times in the day in a small wineglassful of hot water or hot milk. Likewise a jorum (*i.e.* an earthen bowl) of hot onion broth taken at bedtime serves admirably to soothe the air passages and to quicken perspiration after the first feverish stage of influenza or catarrh has passed by. To make this, peel a large Spanish onion and divide it into four parts, then put them into a saucepan with half a saltspoonful of salt, and two ounces of butter and a pint of cold water. Let them simmer gently until quite tender, then pour all into a bowl which has been made hot, dredge a little pepper over, and let the porridge be eaten as hot as it can be taken. The allyl and sulphur in the bulbs, together with their mucilaginous parts, soothe the sore mucous membranes and promote perspiration, whilst other medicinal virtues are exercised at the same time on the animal economy. Provings have been made by medical experts of the ordinary red onion, in order to ascertain what its toxical effects are when pushed to an excessive degree, and it has been found that onions, leeks, and garlic, when taken immoderately, induce melancholy with severe catarrh. They also dispose to sopor, lethargy, and even insanity. The symptoms which appear are extreme watering of the eyes after frequent sneezing, confusion of the head, and heavy defluxion from the nose, with pains in the throat, extending to the ears—in a word, all the accompaniments of a severe cold, "sneezings, lacrymation, pains in the forehead, and a hoarse, hacking cough." These being the result of taking onions in harmful quantities, it is easy to understand that when similar morbid symptoms have arisen spontaneously from other causes, as from an attack of sharp catarrh in the head and chest, modified forms of the onion are likely to act in a beneficial and curative way; on which principle the onion porridge is a philosophical remedy both as food and as physic during the progress of a catarrhal attack, and on the same principle the medicinal tincture of the red onion may be likewise curatively given By eating a few raw parsley leaves immediately afterwards, the strong smell which onions communicate to the breath may be remedied.

*(To be continued.)*

